# Can age‐related changes in parental care modulate inbreeding depression? A test using the burying beetle, *Nicrophorus orbicollis*

**DOI:** 10.1002/ece3.9391

**Published:** 2022-10-05

**Authors:** Matthew Schrader, Parker Hughes, Samuel Jenkins, Ian Kusher, Jonathan Lopez, Harriet Oglesby, Katie E. McGhee

**Affiliations:** ^1^ Department of Biology University of the South Sewanee Tennessee USA

**Keywords:** age, inbreeding by environment interaction, insect, parental investment

## Abstract

Parental care has been shown to reduce the magnitude of inbreeding depression in some species with facultative care. However, parents often vary in the quality or amount of care they provide to their offspring, and it is less clear whether this variation also impacts the magnitude of inbreeding depression. Here, we tested whether age‐related changes in parental care modulate the expression of inbreeding depression in the burying beetle, *Nicrophorus orbicollis*. Consistent with previous studies, we found that older parents produced larger broods of offspring than younger parents without sacrificing mean larval mass. Inbreeding depression was evident in several fitness‐related traits: brood size at dispersal, the proportion of the brood that survived to eclosion, and mean age at death were all reduced in inbred broods compared with outbred broods. Surprisingly, inbred offspring were heavier at dispersal than outbred offspring. This was likely due to reduced sibling competition in inbred broods. Despite evidence for age‐related changes in parental investment and the existence of inbreeding depression, there was no evidence that an interaction between the two influenced any of the traits we measured. Our results suggest that age‐related changes in parental care may be too slight to influence the expression of inbreeding depression.

## Graphical abstract

Here, we tested whether age related changes in parental care modulate the expression of inbreeding depression in the burying beetle, *Nicrophorus orbicollis*. Despite evidence for age related changes in parental investment and the existence of inbreeding depression, there was no evidence that an interaction between the two influenced any of the traits we measured. Our results suggest that age‐related changes in parental care may be too slight to influence the expression of inbreeding depression.

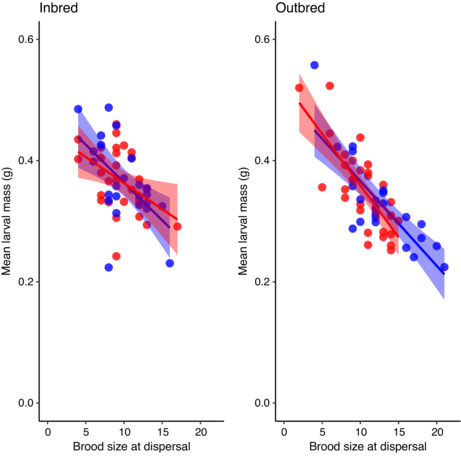

## 1 INTRODUCTION

Inbreeding depression occurs when the offspring of closely related parents have reduced fitness compared with the offspring of unrelated parents. The genetic cause of inbreeding depression is increased homozygosity in the offspring of consanguineous matings. This can reduce fitness through increased homozygosity for recessive deleterious alleles or through decreased heterozygosity for alleles at loci that have overdominant effects on fitness (Charlesworth, 1987; Charlesworth & Willis, 2009). The magnitude of inbreeding depression will depend upon the history of inbreeding within a lineage: Populations with an evolutionary history of inbreeding (e.g., self‐fertilizing hermaphrodites) may have purged recessive deleterious alleles that reduce fitness when homozygous, thus reducing inbreeding depression (Charlesworth et al., 1990; Husband & Schemske, 1996; Schemske & Lande, 1985). It has also been suggested that the magnitude of inbreeding depression may be greater in stressful environments (i.e., those that reduce fitness) than in benign environments (Armbruster & Reed, 2005; Cheptou & Donohue, 2011). Although the impact of the environment on inbreeding depression is variable among taxa and environments, meta‐analyses suggest that inbreeding depression (measured as the median number of lethal equivalents) is generally greater in stressful environments than benign environments and that the magnitude of inbreeding depression increases linearly with the magnitude of environmental stress (Armbruster & Reed, 2005; Fox & Reed, 2011). These results come mostly from laboratory studies where environments are relatively benign and inbreeding coefficients are manipulated experimentally (e.g., by mating full sibs or unrelated individuals). Evidence for environmental dependence of inbreeding depression in the wild (where environments can be harsh and inbreeding coefficients are more variable) is less clear (e.g., Walling et al., 2011).

In animals with parental care, offspring develop in an environment that is created by one or both parents. This “nursery” serves to protect offspring from environmental stressors and is also the site of social interactions between family members (Mock & Parker, 1997; Smiseth et al., 2012). Variation in this environment has consequences for phenotypic development and fitness in many species (Smiseth et al., 2012). For example, competition among dependent siblings within a nursery can influence juvenile growth and ultimately adult body size (Smiseth et al., 2007, 2014). In addition, variation in the amount or quality of care provided by parents can influence offspring phenotype and fitness (Eggert et al., 1998; Steiger, 2013). Given the importance of the nursery environment for offspring fitness, it is possible that the magnitude of inbreeding depression will also depend on the quantity and quality of care that young receive from their parents.

Experimental tests of whether variation in the nursery environment impacts inbreeding depression have only recently been conducted (Meunier & Kölliker, 2013; Pilakouta et al., 2015). These tests have focused on whether parental care can reduce inbreeding depression in species with facultative parental care. In such species, broods of inbred or outbred young can be reared with or without parental care. This allows the magnitude of inbreeding depression to be estimated and compared under different parental care environments. The first experimental study to test whether the presence of parental care affects the magnitude of inbreeding depression focused on the European earwig (*Forficula auricularia*; Meunier & Kölliker, 2013). Females of this species tend eggs and nymphs for several weeks, and parental care involves protecting the brood and providing it access to food (Kölliker, 2007). However, the benefit of maternal care in *F. auricularia* depends on whether food is limiting. When food is restricted (or broods are large), maternal care creates a stressful environment for offspring because mothers and offspring compete with one another for resources and this reduces offspring growth and survival (Meunier & Kölliker, 2012). Meunier and Kölliker (2013) manipulated the presence of maternal care, inbreeding status of the offspring, and brood size in *F. auricularia* and found that maternal presence and inbreeding status interacted to effect nymph development rate: Inbreeding depression in this trait was greatest in the absence of maternal care. However, there was no evidence for inbreeding depression in nymphal survival or recruitment. More recent experiments have tested whether posthatching parental care affects the magnitude of inbreeding depression in the burying beetle, *Nicrophorus vespilloides*. Parental care in this species is more elaborate than in *F. auricularia* and involves pre‐ and posthatching parental care (Royle et al., 2013; Scott, 1998). Prehatching care entails the preparation of a vertebrate carcass that larvae feed from after they hatch. Posthatching care involves direct provisioning of larvae with predigested carrion. Posthatching care in *N. vespilloides* is beneficial but facultative in the sense that larvae can successfully develop without it (Eggert et al., 1998). Experiments with *N. vespilloides* indicate that the presence of parental care reduces the magnitude of inbreeding depression in larval survival to dispersal and posteclosion life span (Pilakouta et al., 2015). There is also evidence that *N. vespilloides* females directly alter their parental care behaviors in ways that alleviate inbreeding depression in their offspring. For example, outbred female parents provided more direct parental care to inbred larvae than outbred larvae and this is associated with increased larval mass (Mattey et al., 2018).

These recent experiments with *N. vespilloides* indicate that the presence of a caring parent can reduce the magnitude of inbreeding depression in offspring and raise the possibility that other parental effects might modulate the magnitude of inbreeding depression. For example, positive correlations between maternal size and investment per offspring have been described in many taxa (Rollinson & Rowe, 2016). These correlations suggest that large parents might invest more in rearing offspring or provide more effective parental care than small parents (Steiger, 2013). If this is the case, then the magnitude of inbreeding depression expressed in offspring might be influenced by parental body size (Pilakouta & Smiseth, 2016). The importance of parental effects might also vary with parental age. For example, while some species show a negative relationship between parental age and offspring fitness consistent with maternal effect senescence (Fay et al., 2016; Ivimey‐Cook & Moorad, 2020), other species appear to become more effective parents as they age (Creighton et al., 2009; Fox et al., 2003; Ivimey‐Cook & Moorad, 2020), and still others show a hump‐shaped relationship between parental age and parental performance (Perrins & Moss, 1974). These age‐related changes suggest that the environment experienced by offspring might vary with parental age and that this might impact the expression of inbreeding depression. Few studies have examined the impact of parental age on inbreeding depression, and these studies have shown mixed results. In the seed beetle, *Callosobruchus maculatus*, the magnitude of inbreeding depression increases with parental age (Fox & Reed, 2010). However, in *Drosophila melanogaster*, the magnitude of inbreeding depression is lower in old parents than it is in young parents (Tan et al., 2013). Both of these species lack parental care; thus, parental age likely impacts inbreeding depression through changes in maternal investment in eggs. It is still unclear whether parental age impacts inbreeding depression in species with parental care, where parents can influence offspring development after hatching.

In this study, we experimentally tested whether parental age influenced the expression of inbreeding depression in the burying beetle *N. orbicollis*, a species with complex posthatching parental care. Like all burying beetles, *N. orbicollis* relies on vertebrate carrion to breed. Parents prepare a carcass by removing the fur or feathers, shaping it into a ball, and smearing it with oral and anal exudates (Royle et al., 2013; Scott, 1998). After hatching, larvae move from the soil to the carcass where they will feed and grow. Larvae are able to self‐feed; however, parents provide posthatching care that involves directly feeding larvae regurgitated carrion (Capodeanu‐Nägler et al., 2016; Royle et al., 2013; Scott, 1998). Posthatching care in *N. orbicollis* is obligate, usually provided by both parents, and beneficial to larvae (Benowitz & Moore, 2016; Capodeanu‐Nägler et al., 2016). Previous work on *N. orbicollis* has also shown that parental investment changes as individuals age. Specifically, older parents increase investment into the current brood and reduce investment into residual reproductive value compared with younger parents (Creighton et al., 2009). This shift is evident as age‐related changes in the number of dispersing larvae and the mass of these larvae. Young parents produce the same initial clutch size as old parents; however, larval survival (measured as the number of larvae at dispersal) is higher when parents are old than when they are young (Creighton et al., 2009). The impact of parental age on mean larval mass is more complicated. When parents breed on small carcasses, mean larval mass is not affected by parental age (old parents produce larger broods of offspring without sacrificing mean larval mass); however, when parents breed on large carcass, old parents produce smaller larvae than young parents (Creighton et al., 2009). These age‐related changes in parental investment provide an excellent opportunity to test whether the magnitude of inbreeding depression changes with parental age.

## 2 METHODS

The beetles in our experiment were part of a laboratory population founded from 100 pairs collected on the campus of the University of the South (Franklin County, TN, USA). The laboratory population had been maintained without inbreeding for eight generations prior to this experiment (breeding 80–100 pairs per generation with no full sibling mating) with additional field‐collected beetles bred into the population at generation six. The goal of our experiment was to test whether parental age influences the magnitude of inbreeding depression in offspring. To do this, we created mating pairs in which parental age and relatedness were factorially manipulated. Mating pairs consisted of either young individuals (14‐day posteclosion) or old individuals (42‐day posteclosion) that were either unrelated or full siblings. All of these adult beetles were virgins prior to being bred in this experiment. We also note that all of the adults were outbred (i.e., they were not the offspring of related individuals).

We assigned individuals to mating pairs 48 h before breeding. In the treatments where parents were unrelated (young parents/nonsib mating and old parents/nonsib mating), individuals were assigned to a pair randomly with respect to family of origin with the only exception that the female and male could not be from the same family. In the treatments where sibs were mated (young parents/sib mating and old parents/sib mating), we chose females randomly with respect to the family and then paired these females with a male sibling. We initially assigned 40 pairs to each treatment; however, our sample sizes were reduced because some beetles died before the beginning of the experiment and others were incorrectly sexed at eclosion. After accounting for this, the initial sample sizes in our treatments were as follows: young parents/nonsib mating = 32, young parents/sib mating = 34, old parents/nonsib mating = 25 old parents/sib mating = 29.

We first observed each pair to test whether parental relatedness (sib or nonsib mating) and parental age affected the likelihood of mating. We placed each pair in a small plastic box (box dimensions: 8.5 cm × 8.5 cm × 4 cm) and watched them for 5 min or until mating was observed. For each pair, mating was scored as either 1 if they mated or 0 if they did not. These observations were made blindly with respect to the experimental treatments.

Immediately after the mating assay, each pair was placed in a plastic breeding box (dimensions: 15 cm × 13.4 cm × 8 cm) filled with ~4 cm of moist soil (Garden Magic Peat) and a thawed mouse carcass (medium white mice, 13–17.99 g, Rodent Pro). Breeding boxes were then placed in a dark cupboard and left undisturbed until 9 days after pairing, after which we began checking each breeding box for dispersing larvae. When >1 larva was observed in the soil away from the carcass, we counted the number of larvae (brood size at dispersal) and weighed the entire brood (brood mass). Pairs that did not have any eggs or larva 10 days after pairing were recorded as breeding failures and were excluded from all subsequent analyses.

After each brood was weighed, we placed the dispersed larvae from each family into a 5 × 5 × 2 cm “eclosion box.” These boxes were subdivided into 25 cells (1 × 1 × 2 cm), and we placed one larva within each cell. The cells were then covered with damp peat, and the box was covered with a weighted plastic lid. Each family was monitored until the adults began to eclose. We recorded the date that each larva eclosed and upon eclosion, we placed all of the surviving adults (*n* = 966) into individual boxes (box dimensions: 8.5 cm × 8.5 cm × 4 cm) containing damp soil and a small amount of ground beef. The individual boxes were randomly assigned to five equally sized groups of ~200 boxes, and all of the boxes in each group were checked once per week to record adult longevity. During each longevity check, we determined whether the individual was alive or dead. We provided surviving beetles with a small amount of ground beef, and if needed, fresh moist soil. For beetles that had died, we recorded the date of death. We continued these checks until 95% of the beetles had died. For each beetle, we calculated lifespan in days as the date of death minus the date of eclosion. Lifespan in days was converted to life span in weeks by dividing by seven and rounding down. We rounded these estimates down because we were only able to check the beetles once per week; thus, the difference between death date and eclosion date will overestimate life span. For each family, we calculated the mean and median week of death.

We first used generalized linear models (with binomial error) to test whether parental relatedness (sib vs. nonsib), parental age (old vs. young), and their interaction influenced the likelihood of mating during the 5‐min trial and whether the pair produced any dispersing larvae. For both of these analyses, the response variables were coded as 0 or 1 to indicate failure or success.

For successful broods, we next tested whether inbreeding status of the offspring (inbred or outbred), parental age (old vs. young), and their interaction influenced several fitness‐related traits. For simplicity, we subsequently refer to these factors as “inbreeding” and “age.” We focused on four traits that have been measured in previous studies of inbreeding depression in *Nicrophorus* beetles: (i) brood size at dispersal; (ii) mean larval mass at dispersal; (iii) survival from dispersal to eclosion; and (iv) posteclosion life span (Pilakouta et al., 2015). Brood size at dispersal is likely influenced by clutch size, hatching success, and larval survival. Our use brood size at dispersal as a measure of larval survival assumes that clutch size is not influenced by inbreeding or parental age. Preliminary and previously published data suggest that these assumptions are reasonable. First, in a preliminary study, we compared mean clutch size between inbred and outbred broods and found no differences between them (mean size of inbred clutches = 14.60 eggs, *n* = 20; mean size of outbred clutches = 14.35 eggs, *n* = 20; *t* = 0.147, *p* = .88). Second, a previous study of *N. orbicollis* found that initial clutch size did not vary with female age (Creighton et al., 2009).

We analyzed the effects of age, inbreeding, and their interaction on each trait using either general or generalized linear models. Brood size and mean larval mass were analyzed using general linear models (with type III sum of squares). Previous studies have found that mean larval mass declines with brood size in burying beetles (e.g., Schrader et al., 2015; Smiseth et al., 2014), and this was also true in our experiment (correlation between brood size and mean larval mass: *r =* −.697, *p* < .0001, *n* = 107). Thus, we also analyzed mean larval mass including brood size at dispersal (mean centered) as a covariate. For this analysis, we initially included all interactions in the model and then removed nonsignificant interactions involving the covariate, beginning with the three‐way interaction (parental age × inbreeding × brood size). We then tested whether survival to eclosion was affected by age, inbreeding, and their interaction using a generalized linear model with a quasi‐binomial error (dispersion parameter = 0.33). For this analysis, we used the cbind function in R to generate a two‐column matrix with the columns giving the numbers of larvae that survived and the number that died for each family. We tested whether adult life span (measured as the mean week of death) was affected by age, inbreeding, and their interaction using a general linear model (with type III sum of squares). We also performed the same analysis using median week of death as the response variable. This analysis yielded nearly identical results to the analysis using mean week of death as the response variable, so we only present the analysis of mean week at death. We note all of these analyses were conducted at the level of family (e.g., each family had a single brood size at dispersal, a single mean mass, a single proportion of larvae surviving to dispersal, and a single mean adult life span). In addition, we retained the interaction between age and inbreeding in all of our models. We did this because we were interested in testing whether the effect of inbreeding depended upon parental age. This is indicated by the age by inbreeding interaction.

Finally, we calculated the magnitude of inbreeding depression (*δ*) for each trait when parents were old and young. Inbreeding depression was calculated as *δ* = (*w*_o_ − *w*_i_)/*w*_o_ where *w*_o_ is the average trait value in outbred larvae and *w*_i_ is the average trait value in inbred larvae. All statistical analyses were performed using R (R Core Team, 2021).

## 3 RESULTS

**FIGURE 1:**
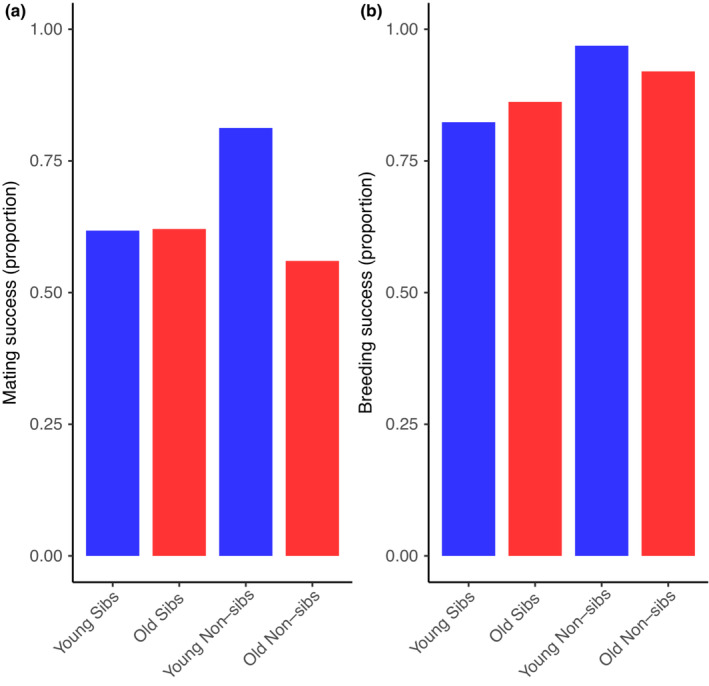
Mating success (a) and breeding success (b) when breeding pairs were young sibs (*n* = 34), old sibs (*n* = 29), young nonsibs (*n* = 32), or old nonsibs (*n* = 23). In each figure, young pairs are indicated in blue, and old pairs are indicated in red. Mating success is the proportion of 5‐min mating trials in which mating was observed. Breeding success is the proportion of pairs that produced at least one dispersing larva.

We observed mating in ~81% of the trials involving young, unrelated adults. In the other treatment combinations, we observed mating in 61%–64% of the trials (Figure 1a). Despite this difference, we found no statistical evidence that parental relatedness (Χ^2^ = 0.86, *p* = .35), age (Χ^2^ = 2.34, *p* = .13), or their interaction (Χ^2^ = 2.45, *p* = .12) influenced whether the beetles mated within 5 min of pairing. The proportion of pairs that produced at least one dispersing larva (Figure 1b) was not related to parental relatedness (Χ^2^ = 3.61, *p* = .057), age (Χ^2^ = 0.24, *p =* .62), or their interaction (Χ^2^ = 0.84, *p* = .36).

**FIGURE 2:**
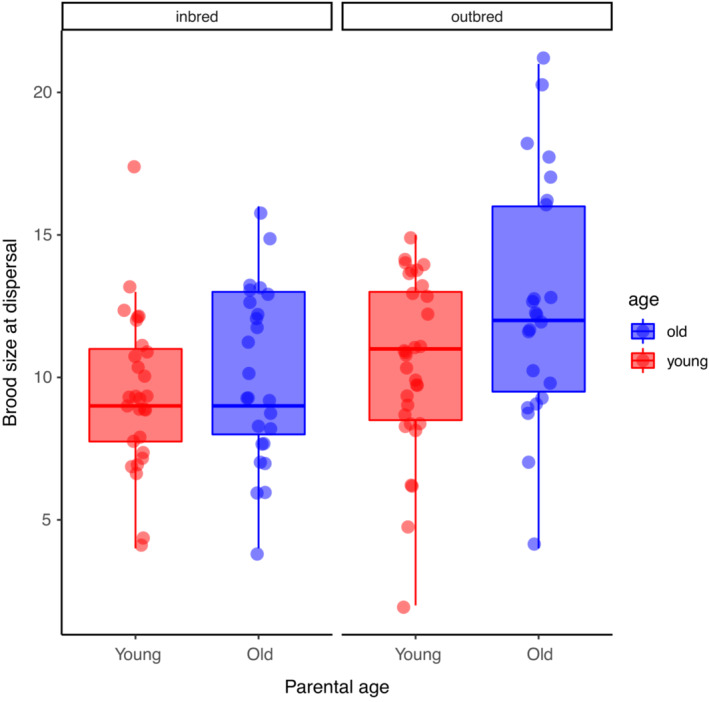
The effects of inbreeding and parental age on brood size at dispersal. For each boxplot, the height of the box represents that interquartile range, the horizontal line is the median, and the whiskers indicate the range. Data points are values for individual broods and are jittered to reduce overlap.

Our next analyses focused only on broods that produced at least one dispersing larvae and were conducted at the level of brood (sample size for each treatment: Young/Inbred = 28; Old Inbred = 25; Young/Outbred = 31; Old/Outbred = 23). We found significant main effects of parental age (*F*_*1,103*_ = 5.35, *p* = .023) and inbreeding on brood size at dispersal (*F*_*1,103*_ = 8.44, *p* = .0045). Old parents produced more dispersing larvae than young parents, and there were more dispersing larvae when larvae were outbred than when they were inbred (Figure 2). However, the interaction between age and inbreeding was not significant (*F*_*1,103*_ = 1.55, *p* = .22; Table 1).

**TABLE 1:** Effects of parental age, inbreeding status of the offspring, and their interaction on several fitness‐related traits

	Age				Inbreeding				Interaction				Covariate			
Trait	*E*_st_	SE	*t*	*p*	*E*_st_	SE	*t*	*p*	*E*_st_	SE	*t*	*p*	*E*_st_	SE	*t*	*p*
Brood size	0.74	0.32	2.312	**.023**	−0.93	0.32	−2.91	**.0045**	−0.40	0.32	−1.25	.22	–	–	–	–
Mean larval mass	0.0029	0.0048	0.59	.55	0.00034	0.0049	0.070	.94	−0.00083	0.0047	−0.17	.86	−0.14	.0014	**−9.33**	<.0001
Survival to eclosion	−0.0049	0.026	−0.19	.85	−0.088	0.026	−3.4	**.0008**	0.0062	0.026	0.24	.81	–	–	–	–
Adult longevity	−0.065	0.11	−0.059	.95	−0.20	0.11	−1.82	.071	−0.027	0.11	−0.24	.81	–	–	–	–

*Note*: We provide the parameter estimates (*E*_st_), standard errors (SE), test statistics (*t* values), and *p* values for each trait. Brood size was analyzed using a two‐way ANOVA, mean larval mass was analyzed using a two‐way ANOVA with brood size (mean centered) as a covariate, survival to eclosion was analyzed using a generalized linear model with quasi‐binomial error, and adult longevity was analyzed using a two‐way ANOVA.

**FIGURE 3:**
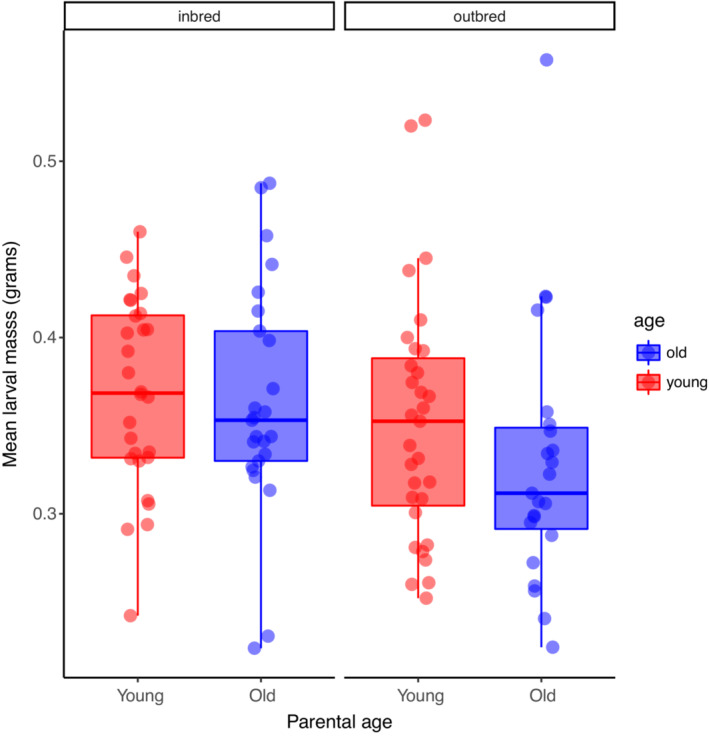
The effects of inbreeding and parental age on mean larval mass For each boxplot, the height of the box represents that interquartile range, the horizontal line is the median, and the whiskers indicate the range. Data points are values for individual broods and are jittered horizontally reduce overlap.

**FIGURE 4:**
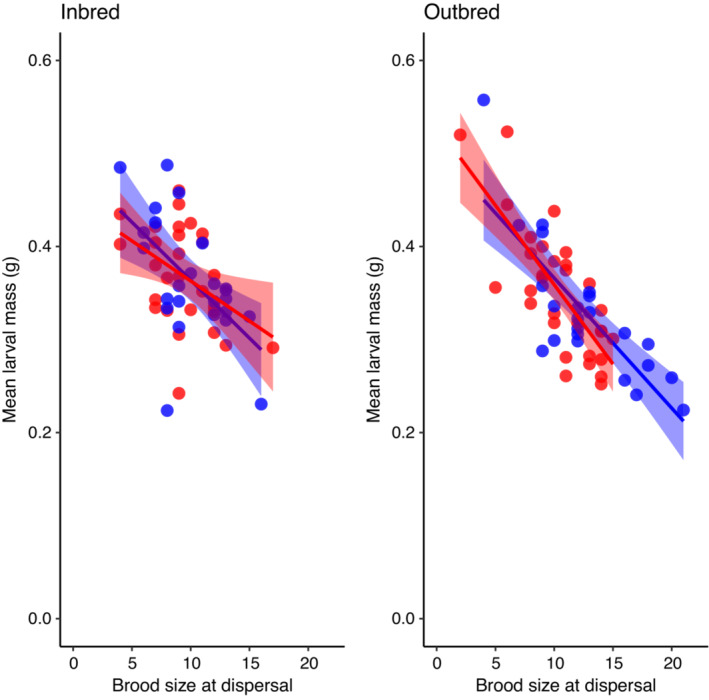
The relationship between brood size at dispersal and mean larval mass for inbred and outbred broods (left and right panels respectively). In ach panel, broods produced by old parents are in blue and broods produced by young parents are in red.

Inbred larvae were significantly heavier at dispersal than outbred larvae (*F*_*1,103*_ = 4.10, *p* = .045; Figure 3); however, mean larval mass was not affected by parental age (*F*_*1,103*_ = 1.25, *p* = .27) or the age by inbreeding interaction (*F*_*1,103*_ = 0.51, *p* = .48). When we included brood size at dispersal as a covariate in this model, we found a strong effect of brood size (*F*_*1,102*_ = 87.02, *p* < .0001) and no effects of parental age (*F*_*1,102*_ = 0.35, *p* = .55), inbreeding status (*F*_*1,102*_ = 0.0049, *p* = .94), or their interaction (*F*_*1,102*_ = 0.030, *p* = .86; Table 1; Figure 4). This suggests that the large size of inbred larvae was driven by the fact that they came from relatively small broods.

**FIGURE 5:**
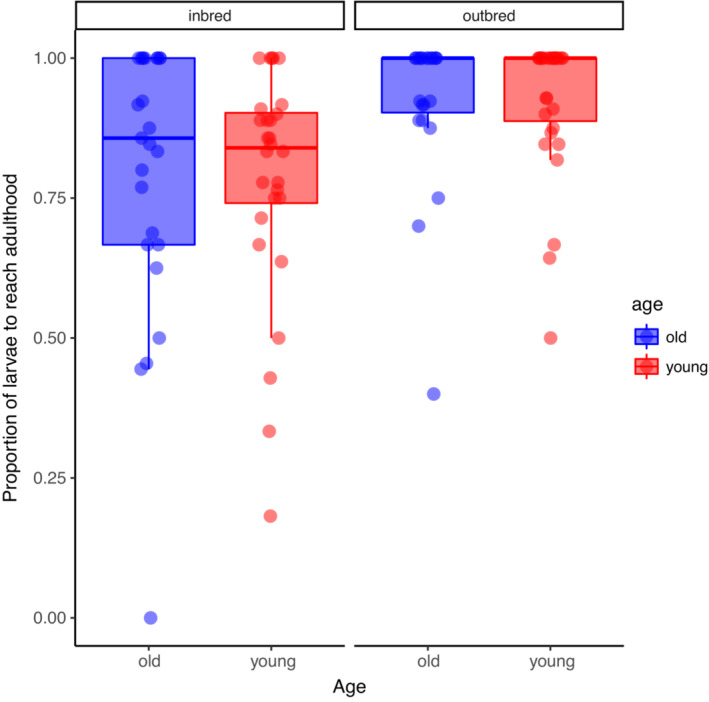
The effects of inbreeding and parental age on survival from dispersal to eclosion. For each boxplot, the height of the box represents that interquartile range, the horizontal line is the median, and the whiskers indicate the range. Data points are values for individual broods and are jittered horizontally to reduce overlap.

**FIGURE 6:**
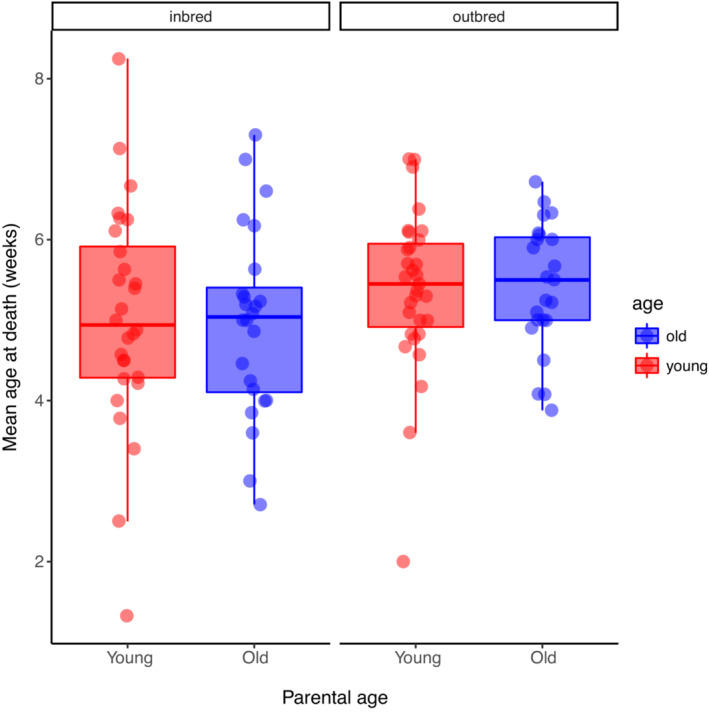
The effects of inbreeding and parental age on mean age at death. For each boxplot, the height of the box represents that interquartile range, the horizontal line is the median, and the whiskers indicate the range. Data points represent the mean age at death (in weeks) for individual families and values are jittered horizontally to reduce overlap.

The proportion of dispersing larvae that survived to eclosion was influenced by inbreeding (Χ^2^ = 11.78, *p* = .0005), with inbred larvae having lower survival to eclosion than outbred larvae (Figure 5). However, survival to eclosion was not influenced by parental age (Χ^2^ = 0.037, *p* = .85) or the age by inbreeding interaction (Χ^2^ = 0.060, *p* = .81;Table 1). Adult longevity was not influenced by inbreeding (*F*_*1,102*_ = 3.32, *p* = .071), parental age (*F*_*1,102*_ = 0.0035, *p* = .95), or the interaction between inbreeding and age (*F*_*1,102*_ = 0.059, *p* = .81; Table 1; Figure 6).

Overall, we found evidence for inbreeding depression (*δ* > 0) in brood size at dispersal, survival from dispersal to eclosion, and adult longevity. The magnitude of inbreeding depression was similar regardless of parental age (Table 2), consistent with the absence of any age by inbreeding interactions in our statistical analyses (Table 1). We also note that the effect sizes (partial *η* ^2^) for the age by inbreeding interactions in our analyses are all very small (*η* ^2^ between 2.97 × 10^−4^ and 0.01; Table S1). These values indicate that the age by inbreeding interaction accounts for <1% of the variation in the traits we measured after accounting for the main effects of inbreeding and age.

**TABLE 2:** The magnitude of inbreeding depression (*δ*) for each trait when parents were young or old

Trait	*δ*_young parents_	*δ*_old parents_
Brood size	0.097	0.209
Mean larval mass	−0.0477	−0.106
Survival from dispersal to eclosion	0.155	0.137
Mean age at death	0.065	0.084

*Note*: Inbreeding depression for mean larval mass was calculated using mean mass for each treatment combination unadjusted for variation in brood size.

## 4 DISCUSSION

In species with parental care, aspects of the family environment exert an important influence on phenotypic development and may also be important in modulating the expression of inbreeding depression. For example, a major function of parental care is to buffer offspring from environmental stress. If parental care reduces stress experienced by offspring, then parental care might alleviate inbreeding depression. Consistent with this, parental care has been shown to reduce the magnitude of inbreeding depression in some species with facultative care (Mattey et al., 2018; Meunier & Kölliker, 2013; Pilakouta et al., 2015). However, parents often vary in the quality or amount of care they provide to their offspring (Steiger, 2013), and it is less clear whether this type of variation also impacts the magnitude of inbreeding depression (Pilakouta et al., 2016; Pilakouta & Smiseth, 2016). Here, we tested whether parental age modulates the expression of inbreeding depression in *N. orbicollis*, a species in which parental investment has been shown to increase with parental age (Creighton et al., 2009). We found that older parents produced larger broods of offspring than younger parents without sacrificing mean larval mass. Although our experiment does not reveal the mechanism through which this happens, it is consistent with the age‐related changes in parental investment described by Creighton et al. (2009). We also found evidence for inbreeding depression in several fitness‐related traits: Brood size at dispersal and the proportion of the brood that survived to eclosion were both reduced in inbred broods compared to outbred broods. Despite evidence for age‐related changes in parental investment and the existence of inbreeding depression, we found no evidence that an interaction between the two influenced any of the traits we measured.

Previous studies of *Nicrophorus* beetles have found evidence for inbreeding‐by‐environment interactions (I × E) when larvae are reared with or without parental care (Pilakouta et al., 2015) and when they are reared by large or small mothers (Pilakouta & Smiseth, 2016). Other experimental manipulations of the nursery environment have yielded mixed results. For example, the level of sibling competition has a large effect on mean larval mass but does not impact the magnitude of inbreeding depression in *N. vespilloides* (Pilakouta et al., 2016). And in our study, parental age did not impact the magnitude of inbreeding depression. It is possible that these variable effects of the nursery environment on the magnitude of inbreeding depression are related to how stressful the family environment is. Fox and Reed's (2011) meta‐analysis of I × E suggests that stress increases the magnitude of inbreeding depression. However, low levels of stress (i.e., those that reduce the fitness of outbred individuals by <25%) have small and idiosyncratic impacts on the magnitude of inbreeding depression (Fox & Reed, 2011). In our experiment, old females produced broods that were about 20% larger than young females; thus, it is likely that the age‐related changes in parental performance that we observed are simply too weak to modulate inbreeding depression.

Although parental age did not influence the magnitude of inbreeding depression, there was evidence that the nursery environment influenced the expression of inbreeding depression in mean larval mass. We found that inbred larvae were actually heavier at dispersal than outbred larva (Figure 3); however, when we included brood size at dispersal as a covariate in this analysis, the effect of inbreeding status became nonsignificant (Figure 4). This suggests that the inbred larvae were heavier because they came from relatively small broods. Previous studies of *Nicrophorus* beetles have shown that mean larval mass declines with brood size (Monteith et al., 2012; Schrader et al., 2015; Smiseth et al., 2014), likely due to increased competition and reduced access to parental care in large broods. In our experiment, it appears that low survival of inbred larvae reduced competition among the surviving brood mates which allowed them to attain a larger body size at dispersal. This is broadly similar to the pattern in Great Tits (*Parus major*), where low hatching success in inbred clutches appears to reduce competition among nestlings resulting in a slightly higher rate of recruitment of inbred offspring compared with outbred offspring (van Noordwijk & Scharloo, 1981). In our experiment, it is unclear whether being large actually increases the fitness of inbred larvae. Previous studies of *Nicrophorus* beetles have shown that large individuals win contests, are better parents, and are better able to take advantage of large carcasses than small parents (Otronen, 1988; Schrader et al., 2022; Steiger, 2013). In our study, reduced competition in inbred broods did not lead to increased survival: inbred broods had lower survival from dispersal to eclosion and a shorter adult life span (although the impact on life span was not statistically significant). Since we did not weigh individual larvae, we are unable to test whether there is an association between an individual's mass and whether it survives to adulthood. It would be interesting in future studies to test whether there is a relationship between larval mass and survival to adulthood and whether this relationship varies with inbreeding status.

The magnitude of inbreeding depression varies considerably both among and within species. Variation in inbreeding depression among species is often attributable to differences in their evolutionary history of inbreeding (Schemske & Lande, 1985). Within populations, variation in inbreeding depression can be attributed to genetic variation (e.g. Pray & Goodnight, 1995) and/or environmental variation (Armbruster & Reed, 2005; Fox & Reed, 2011). Parental age, which can vary considerably within populations, has been suggested as one environmental factor that might explain variation in inbreeding depression (Fox & Reed, 2010; Tan et al., 2013). Although studies of species without parental care have supported this idea (Fox & Reed, 2010; Tan et al., 2013), few studies have tested whether age‐related changes in parental care modulate inbreeding depression. Our results suggest that parental age does not influence the magnitude of inbreeding depression in a species with age‐related changes in parental care. It may be that there is a nonlinear relationship between parental age and inbreeding depression or that a relationship between parental age and inbreeding depression is only apparent across a broader range of inbreeding coefficients (Fox & Reed, 2010). Examining these possibilities will be crucial for better understanding whether and how parental age influences inbreeding depression.

## AUTHOR CONTRIBUTIONS

**Matthew Schrader:** Conceptualization (equal); data curation (equal); formal analysis (equal); project administration (equal); writing – original draft (lead); writing – review and editing (lead). **Parker Hughes:** Formal analysis (equal); investigation (equal); writing – review and editing (supporting). **Samuel Jenkins:** Formal analysis (equal); investigation (equal); writing – review and editing (supporting). **Ian Kusher:** Formal analysis (equal); investigation (equal); writing – review and editing (supporting). **Jonathan Lopez:** Formal analysis (equal); investigation (equal); writing – review and editing (supporting). **Harriet Oglesby:** Formal analysis (equal); investigation (equal); writing – review and editing (supporting). **Katie E. McGhee:** Conceptualization (equal); data curation (equal); formal analysis (equal); investigation (equal); project administration (equal); writing – original draft (supporting); writing – review and editing (supporting).

## CONFLICT OF INTEREST

The authors have no conflict of interest.

## Supporting information


Table S1

## Data Availability

All data are deposited in DRYAD (https://doi.org/10.5061/dryad.dz08kps13).
