# Supplementary material for: Can age‐related changes in parental care modulate inbreeding depression? A test using the burying beetle, Nicrophorus orbicollis
Source: Ecol Evol. 2022 Oct 5;12(10):e9391. doi: 10.1002/ece3.9391 (PMC9534728; doi:10.1002/ece3.9391)
Supplement: Supplementary file 1 — Table S1 [file ECE3-12-e9391-s001.docx]

Table S1. The effect size (partial *η^2^*) of the inbreeding by age interaction for each of the traits we measured and the sample size (n) required for the observed effect size to be significant (α=0.05) with a power of 0.8. Partial *η^2^* is calculated for each trait as SS _interaction_ / (SS _interaction_ +SS _error_). It can be interpreted as the proportion of variance explained by the interaction with the main effects factored out. Effect sizes were obtained for each model using the *effectsize* package (Ben-Shachar and Makowsik 2020) and power analyses were performed using the *pwr* package (Champely et al. 2020).

| **Trait** | $\boldsymbol{\delta}$***η^2^*** | **n** |
| --- | --- | --- |
| Brood size | 0.01 | 788 |
| Mean larval mass | 2.97x10^-4^ | 26433 |
| Survival from dispersal to eclosion | 9.92x10^-3^ | 796 |
| Mean age at death | 5.79x10^-4^ | 13560 |
